# Rap1A promotes ovarian cancer metastasis via activation of ERK/p38 and notch signaling

**DOI:** 10.1002/cam4.946

**Published:** 2016-11-04

**Authors:** Lili Lu, Jingshu Wang, Yougen Wu, Ping Wan, Gong Yang

**Affiliations:** ^1^ Cancer InstituteFudan University Shanghai Cancer CenterDepartment of OncologyShanghai Medical SchoolFudan UniversityShanghai200032China; ^2^ Department of BiologyCollege of Life and Environmental SciencesShanghai Normal University100 Guilin RoadShanghai200234China; ^3^ Central Laboratory, The Fifth People's Hospital of Shanghai, Fudan UniversityShanghai200240China; ^4^ Department of Gynecological Oncology, The Fifth People's Hospital of ShanghaiFudan UniversityShanghai200240China

**Keywords:** ERK/p38, metastasis, Notch, ovarian cancer, Rap1A

## Abstract

As one of the Ras‐associated proteins, Rap1A has been linked to cancer initiation and development. However, the precise function of Rap1A in ovarian cancer is still not understood. Here, we show that Rap1A promotes ovarian cancer tumorigenesis and metastasis via stimulating cell proliferation, migration and invasion both in vivo and in vitro. Mechanistic study showed that Rap1A activates extracellular signal‐regulated kinase (ERK), p38 mitogen‐activated protein kinase (MAPK) and Notch pathways, leading to the enhanced expression of several epithelial‐mesenchymal transition (EMT) markers such as slug, zeb1, vimentin, fibronectin, and MMP9. However, the pretreatment of Rap1A‐overexpressing cells with the Notch inhibitor DAPT or ERK inhibitor (U0126) inhibited the up‐regulated expression of those molecules. These findings provide the first evidence linking Rap1A with ovarian cancer development through the ERK/p38 and Notch signaling pathways, indicating that Rap1A may be used as a novel diagnostic marker or a therapeutic target for ovarian cancer.

## Introduction

Ovarian carcinoma is the most lethal gynecological malignancy, which ranks the fifth leading cause of cancer death among women by 2016 1. Since the symptoms are not obvious at the early stage, many patients are diagnosed at advanced stage with poor prognosis 2. For patients whose tumors remain sensitive to the platinum‐based chemotherapy and paclitaxel, their life span could be extended until the chemoresistance occurs 3. Therefore, it becomes an urgent demand to identify novel biomarkers for precise diagnosis, efficient treatment, and predictive prognosis of ovarian cancer patients.

Rap1A is a member of the Ras‐associated proteins (Raps) bearing the highest resemblance to Ras 4, 5. Similar to other Raps, Rap1A switches between the GTP‐bound and GDP‐bound states and stimulates downstream effectors to deliver signals and to make functions 6, 7. After two decades of study, it is now believed that Rap1A plays a vital role in regulation of extracellular signal regulated kinase (ERK) activation and integrin‐mediated cellular activities, and is closely related to cell proliferation, adhesion, cell metastasis as well as osteoblast differentiation 5, 8, 9, 10. However, the specific role of Rap1A varies in different cell types. In glioblastoma, researchers demonstrated that Rap1A promotes glioblastoma proliferation and tumor growth 11. Knocking down of Rap1A with miRNAs inhibited the Rac1/PAK1 pathway and attenuated prostate cancer cell proliferation, adhesion, and invasion 12, 13. These results suggest that Rap1A may exert its effect as an oncogene. Herein, we investigated the role of Rap1A in some ovarian cancer cell lines and tried to uncover the Rap1A associated mechanism in ovarian cancer.

The epithelial‐mesenchymal transition (EMT) was initially found to be associated with embryonic development, chronic inflammation, or tissue reconstruction 14. EMT program also owns traits like the acquisition of motility and invasion, which enables carcinoma cells to favor migration and invasion 15. In multiple cell types, the transcription factors (TFs) such as slug, snail, zeb1 induced by diverse extracellular signals may orchestrate EMT programs. Several studies have reported that EMT is of vital importance in the development of ovarian cancer 16, 17, 18.

In this study, by silencing or overexpression of Rap1A and drug treatment, we investigated the effects of Rap1A on ovarian cancer cell proliferation, invasion, and metastasis. We found that Rap1A functions as an oncogene to promote ovarian cancer metastasis through activation of the ERK/p38 and Notch signaling.

## Materials and Methods

### Cell lines and cell culture

Human epithelial ovarian cancer cell lines HEY, HEYA8, OVCA429, OVCA420, A2780, SKOV3 with p53 null, and lentivirus packaging cells (293T) were purchased from American Type Culture Collection (Manassas, VA). HEY, HEYA8, OVCA429, OVCA420, A2780, SKOV3 cells were cultured in Roswell Park Memorial Institute (RPMI) 1640 medium and 293T cells were maintained with Dulbecco's Modified Eagle's Medium (DMEM). All cells were kept under conditions recommended by the suppliers.

### Plasmids construction, cell transfection and viral infection

To enhance the expression of RAP1A in HEYA8, human wild type Rap1A cDNAs labeled HA‐tag was inserted into PCDH‐CMV‐MCS‐EF1‐PURO. Viruses produced from 293T cells transfected with PCDH were collected to infect HEYA8 cells and to establish the Rap1A overexpression cell line, using the previously published methods 19. The control cell line was generated by infection with viruses containing empty vector. The resulting cells were selected with puromycin (1.5–2.0 *μ*g/mL) for 7–14 days.

The DNA oligonucleotides were designed to generate shRNAs against the open reading frame of mRNA 5'‐ GCTCAGT CTACGTTTAATGAT‐3' (Rap1A shRNA1) and 5'‐ CCGAGCAATTTACAGCAATGA ‐3' (Rap1A shRNA2) to knockdown the expression of Rap1A. PLKO.1/Rap1A shRNA was generated according to the previously reported method.^20^. The control vector was similarly constructed by directly inserting a scrambled shRNA (Scr sh) into PLKO.1. New cell lines including HEY‐Rap1A shRNA and SKOV3‐Rap1A shRNA were generated, using the method mentioned above. Corresponding control cell lines were made by infection of viruses expressing the scrambled shRNA (Scr sh). The infected cells were selected with puromycin (1.5–2.0 *μ*g/mL) for 7–14 days. The resulting cells were used for following experiments without addition of puromycin.

### Cell proliferation

Cells were detached using trypsinization and washed twice with PBS. 2 × 10^3^ cells per well were seeded in 96‐well culture plates (Corning Inc., Corning, NY) in 100 *μ*L medium and cultured for 1,3,5,7and 9 days. Cell growth was detected, using 5 mg/mL MTT solution (sigma) according to the manufacturer's instructions. The OD at 490 nm was quantified using a Tecan Infinity 200PRO multi‐well plate reader (Tecan Ltd., Switzerland). The assay was independently repeated three times.

### Cell invasion and migration

To identify cell invasion ability, we used a high throughput screening multi‐well insert 24‐well two‐chamber plate (BD Biosciences, San Jose, CA), with an 8‐*μ*m (pore size) polycarbonate filter between chambers. 2.5 × 10^4^ cells of nA8‐Rap1A cDNA, HEY‐Rap1A sh1, SKOV3‐Rap1A sh2, and their corresponding controls were placed into the upper chamber and permitted to invade at 37°C for 48 h toward a lower reservoir containing medium and coated with Matrigel (BD Biosciences). The chambers were then fixed in 100% methanol for 30 min and stained with crystal violet for 10 min. The invasive cells which passed through the membrane were counted at ×200 magnification with five representative fields under a microscope. For migration, 2.5 × 10^4^ cells were added into the upper chamber without Matrigel and kept for 36 h under regular conditions. All the above assays were repeated in triplicate.

Scratch assay was performed to examine cell migration speed. Cells were incubated in 6‐well plate over‐night to yield monolayer confluence. By scratching with a pipette tip and photographing immediately (time 0) and 12 h' later, the distance migrated by the cell monolayer to close the scratch area during the time period was observed and measured. The ratio of the cell migration distance at 12 h to that at 0 h was analyzed as the migration index. The assay was carried out in triplicate and repeated three times.

### Anchorage‐independent colony formation

According to the previously published method 21, cells stably expressing shRap1A and Rap1A cDNA were used to perform soft agar assay. Briefly, 5 × 10^4^ cells were suspended with 0.35% agarose (Life Technologies) in 1640 containing 10% FBS, and the suspension was laid on top of 5 mL of solidified 0.7% agarose. Triplicate cultures of each cell type were maintained for 14–28 days at 37°C in a 5% CO_2_ atmosphere, and fresh medium was fed every 7 days. Only colonies over 50 *μ*m (~100 cells) in diameter in each dish was counted at 14–20 days. The assay was repeated three times in duplicate.

### Immunoblotting analysis

To analyze Rap1A expression in cells, we prepared cell lysates at 75% of confluence using 500 *μ*L of radioimmunoprecipitation assay buffer (RIPA, 25 mmol/L Tris–HCl at pH 7.6, 150 mmol/L NaCl, 1% Nonidet P‐40, 1% sodium deoxycholate, and 0.1% sodium dodecyl sulfate). Protein concentrations of the lysates were determined with a Bio‐Rad protein assay kit (Hercules, CA). Immunoblotting analyses were performed as described previously. Antibodies against the following proteins were obtained from Santa Cruz Biotechnology: Rap1A, GAPDH. Antibodies against the following proteins were from Cell Signaling Technology (Danvers, MA): MEK1/2, ERK1/2, p38, pMEK1/2 (Ser217/221), pERK1/2 (Thr202/Tyr204), and pp38 (Thr180/Tyr182). The secondary antibodies were F(ab)2 fragment of donkey anti‐mouse immunoglobulin (product NA931) or of donkey anti‐rabbit immunoglobulin (product NA9340) linked to horseradish peroxidase from Amersham Biosciences (Little Chalfont, Buckinghamshire, UK). Immunoblotting reagents were from an electrochemiluminescence kit (Amersham Biosciences). Cells treated with U0126 (ERK inhibitor, Sigma) at indicated concentrations were also analyzed, using the above methods.

### Xenograft tumors in nude mice

The animal experiments were approved by the Institutional Animal Care and Use Committee of ECNU (East China Normal University, ECNU) and performed following the Institutional Guidelines and Protocols. Xenograft tumors were either subcutaneously to detect tumor growth or intraperitoneally to monitor tumor metastasis of HEY‐Rap1A sh1, and HEYA8‐Rap1A cDNA cells and their controls.

5 × 10^6^ cells of each cell line mentioned above were bilaterally injected into 6‐week‐old female BALB/c athymic nude mice (offered by Slac Laboratory Animal (Shanghai, China). Each cell line was injected into three mice for a total of six injections. Subcutaneous tumors were measured with a vernier caliper every 3 days. When a tumor reached 1.0 cm in diameter, the mouse was sacrificed and the tumors were weighed and measured. The longest diameter “a” and the shortest diameter “b” of tumors was measured, and we calculated the tumor volume with the formula: V (in mm^3^) = a × b^2^ × 0.52 22, where 0.52 is a constant to calculate the volume of an ellipsoid.

Six mice were chosen to be injected with 1 × 10^7^ cells of each cell lines intraperitoneally, and mice were observed regularly and sacrificed before natural death occurred. Tumor nodules were removed, counted, and the mice were weighed.

### Cell spheroid formation

For preparation, we diluted material (corning #354236) to 50 *μ*g/mL with 0.02N acetic acid, added 300–500 *μ*L diluted material into 24‐well plate and incubate at room temperature for 1 h. After that, the remaining solution should be carefully aspirated, using PBS rinse well to remove acid for 2–3 times. Then the needed number of cells (250–300) and the appropriate volume of 1640 medium were mixed to make the cell concentration at 250 cells/500 *μ*L in one tube. Another tube contained the 1640 medium, material and NaOH at the ratio of 5:1:0.0235. Mixed two tubes on ice and then plated the mixture in the well. After one‐week incubation, spheroid numbers were counted under a phase‐contrast microscope. Cells treated with DAPT (Notch inhibitor, Sigma #D5942) at the indicated concentrations were also used for spheroid formation assay.

### Statistical analysis

All data were analyzed by the Student *t* test. *P *<* *0.05 was considered statistically significant.

## Results

### Rap1A increases cell proliferation of ovarian cancer

**Figure 1:**
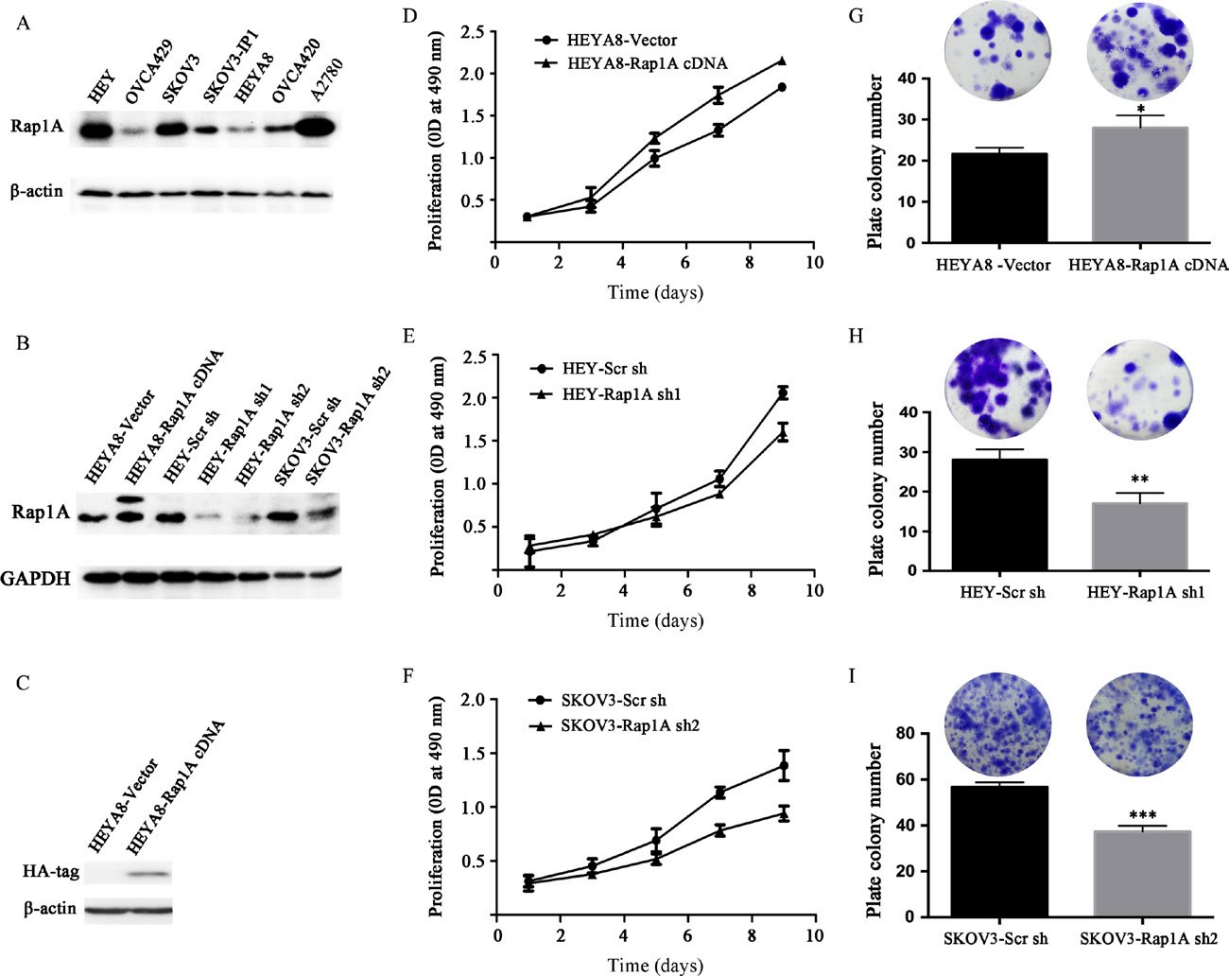
Rap1A increases cell proliferation. (A) Detection of Rap1A in ovarian cancer cell lines. *β*‐actin is a loading control. (B) Analyses of Rap1A in cells. GAPDH is a loading control. (C) Detection of HA‐tag in HEYA8‐vector and HEYA8‐Rap1A cDNA cells. *β*‐actin is a loading control. (D) Cell viability was determined by the MTT in cells at the indicated time. (E) Colony‐forming capability of cancer cells was measured, using a colony formation assay after incubation for 15 days. Representative wells are shown in the upper panel, and quantitative analysis of the relative colony number for each group is shown in the lower panel. Values are expressed as the mean ± standard deviation (*n* = 3wells). *P < 0.05 vs. the control. **P < 0.01 vs. the control. ***P < 0.005 vs. the control.

To investigate the function of Rap1A in human ovarian cancer, we first detected the expression level of Rap1A in various human ovarian cancer cell lines. We found that the expression of Rap1A was higher in HEY, SKOV3, and A2780 cells than in OVCA429 and HEYA8 cell lines (Fig. 1A). So HEY and SKOV3 cells were chosen to establish Rap1A silencing cell lines (HEY‐Rap1A sh1 and SKOV3‐Rap1A sh2). Corresponding control cell lines were established with lentivirus expressing scrambled shRNA (Scr sh). As tested by Western blotting, the protein level of Rap1A in HEY and SKOV3 cells were markedly reduced compared with in control cells (Fig. 1B). Meanwhile, HEYA8 cells expressing the Rap1A cDNA plus HA‐tag appeared with a higher band tested with antibody to Rap1A (Fig. 1B), which was also detected by antibody against HA (Fig. 1C). We next evaluated the cell proliferation rate by MTT assay. The proliferation rates of Rap1A shRNA‐treated HEY and SKOV3 cells were much lower than the corresponding control cells (Fig. 1E,F). Rather, HEYA8 cells expressing Rap1A cDNA had a higher proliferation rate than control cells (Fig. 1D). Consistently, a colony formation assay showed that the number of colonies formed by HEYA8‐Rap1A cDNA was significantly increased (Fig. 1G), while the colony number formed by HEY‐Rap1A sh1, or SKOV3‐Rap1A sh2 cells were obviously decreased compared with those formed by controls (Fig. 1H, I).

### Rap1A promotes ovarian cancer cell migration and invasion

**Figure 2:**
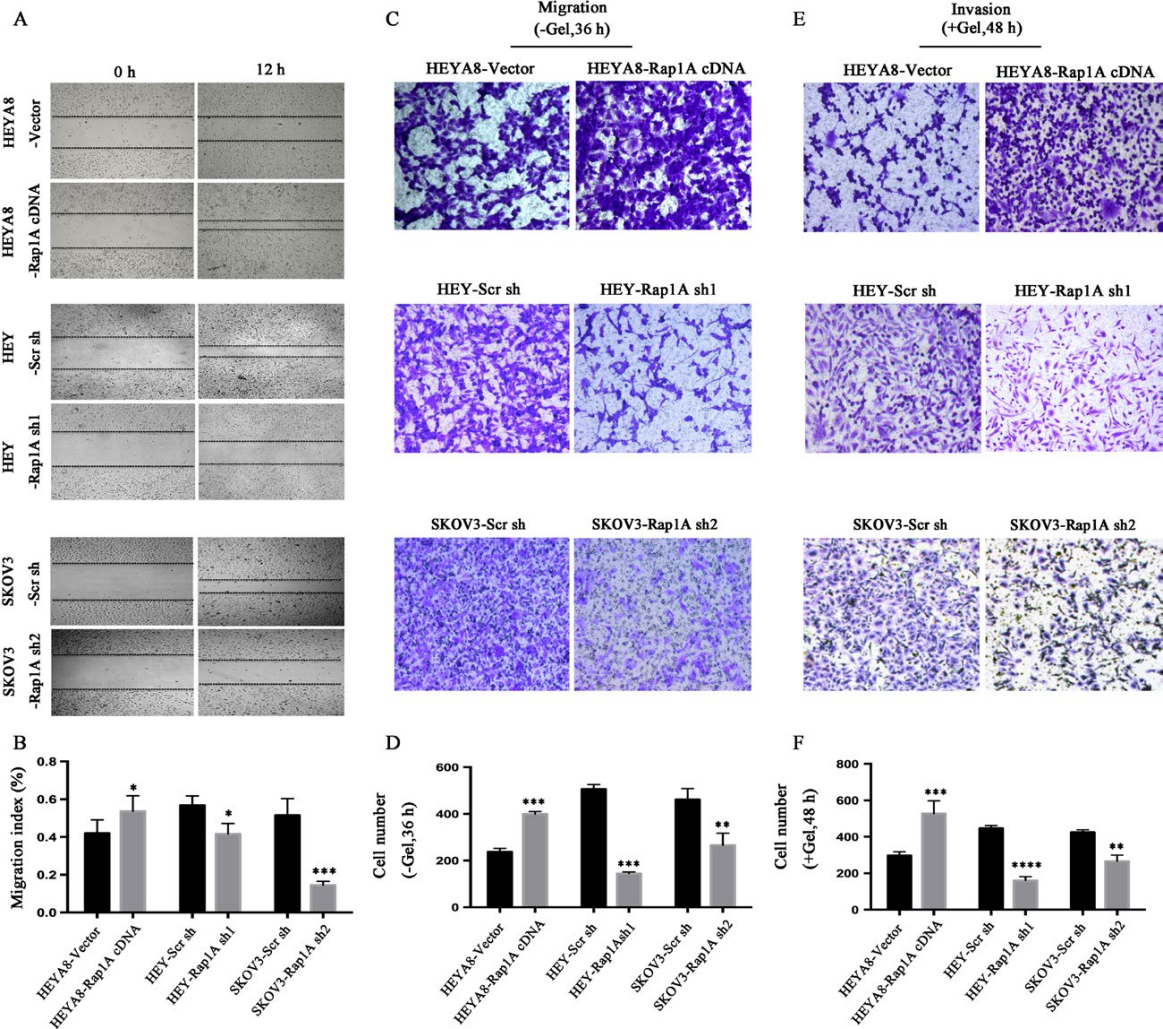
Rap1A promotes migration and invasion. (A) Detection of migration speed of cancer cells at indicated time, compared with their control cells, respectively, *n* = 3 wells. (B) Quantitative analysis of above mentioned migrated cells (*P < 0.05 vs. the control; ***P < 0.005 vs. the control.). Error bars = 95% CIs. (C) Microscopy images of migrated cells by cancer cells and their controls after incubation in permeable supports for 36 h, staining with crystal violet (magnification, 10×). (D) Quantification of migrated cells. (**P < 0.01 vs. the control; ***P < 0.005 vs. the control.). Error bars = 95% CIs. *N* = 3 wells. (E) Microscopy images of migrated cells after incubation 48 h in permeable supports toward a lower reservoir containing medium plus Matrigel, staining with crystal violet (magnification, 10×). (F) Quantification of invaded cells. (**P < 0.01 vs. the control; ***P < 0.005 vs. the control.). Error bars = 95% CIs. *n* = 3 wells.

We then detected the effects of Rap1A on cell migration and invasion. By scratch assay we found that the migration speed of HEYA8‐Rap1A cDNA cells was accelerated after 12 h culture, but the migration speed of HEY‐Rap1A sh1 or SKOV3/Rap1A sh2 cells was slowed down after 12 h culture compared with those of control cells (Fig. 2A, B). Thus, Rap1A overexpression stimulated cell migration, while Rap1A silencing inhibited cell migration (Fig. 2C, D). Additionally, the results from Figure 2 also showed that the number of invaded cells expressing Rap1A cDNA was highly increased, whereas the number of invaded cells expressing Rap1A shRNA was much reduced, compared with those of control cells (Fig. 2E, F). Therefore, Rap1A may function to promote ovarian cancer cell migration and invasion.

### Rap1A enhances tumor sphere via activation of notch signaling

**Figure 3:**
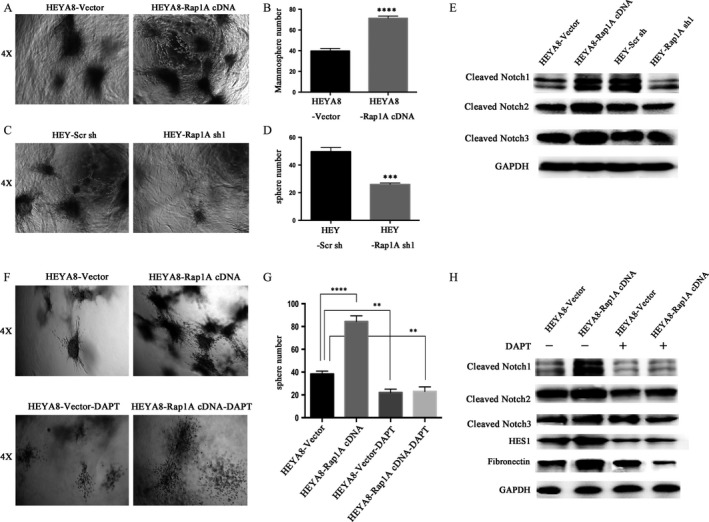
Sphere formation. (A,C) Cancer cells were implanted in rat‐tail gels for about 7 days. (magnification, 4×). (B, D) Quantification of the tumor sphere number in cancer cells. ***P < 0.005; ****P < 0.0001vs. the control; *n* = 3. (E) Detection of cleaved NOTCH1, NOTCH2 and NOTCH3 in cells. GAPDH is a loading control. (F) The tumor sphere formation efficiency of cells with or without DAPT treatment was detected by tumor sphere assay. (magnification, 4×). (G) Quantification of the tumor sphere number in cells with or without the treatment of DAPT. **P < 0.01. ****P < 0.0001vs control; *n* = 3. (H) Analysis of cleaved NOTCH1, NOTCH2, NOTCH3, HES1, and Fibronectin in HEYA8‐vector and HEYA8‐Rap1A cDNA cells treated with or without DAPT. GAPDH is a loading control.

As a surrogate assay for tumor‐initiating ability, we tested the formation of cell spheres in three‐dimensional cultures at clonal densities that recapitulate the function of Rap1A in cell self‐renew and proliferation to closely mimic the in vivo physiological situation that favors stem cell growth. We observed a significant increase of sphere numbers and sizes by overexpression of Rap1A in HeyA8 cells (Fig. 3A, B), whereas silencing of Rap1A inhibited cell sphere formation (Fig. 3C, D). Moreover, we found that the expression of the cleaved Notch1, the cleaved notch2 and notch3 protein levels were either attenuated or enhanced in shRNA or cDNA expressing cells compared with in control cells (Fig. 3E). We also determined whether blocking the Notch signaling with the *γ*‐secretase inhibitor DAPT was able to inhibit ovarian cancer cell migration. We treated tumor cells with DAPT at the concentrations of 20 *μ*mol/L for 24 h. As shown, pretreatment of HeyA8‐vector and HeyA8‐Rap1A cDNA cells with DAPT decreased the number of spheroids by 50% compared with control cells (Fig. 3F, G). The cleaved Notch1, 2, 3 protein levels as well as notch target gene Hes1 expression were highly reduced in both HEYA8‐vector and HEYA8‐Rap1A cDNA cells following treatment with DAPT compared with in DMSO‐treated control cells (Fig. 3H). Thus, Rap1A may positively regulate the Notch signaling to promote ovarian cancer cell initiation.

### Rap1A accelerates ovarian cancer tumorigenesis and metastasis

**Figure 4:**
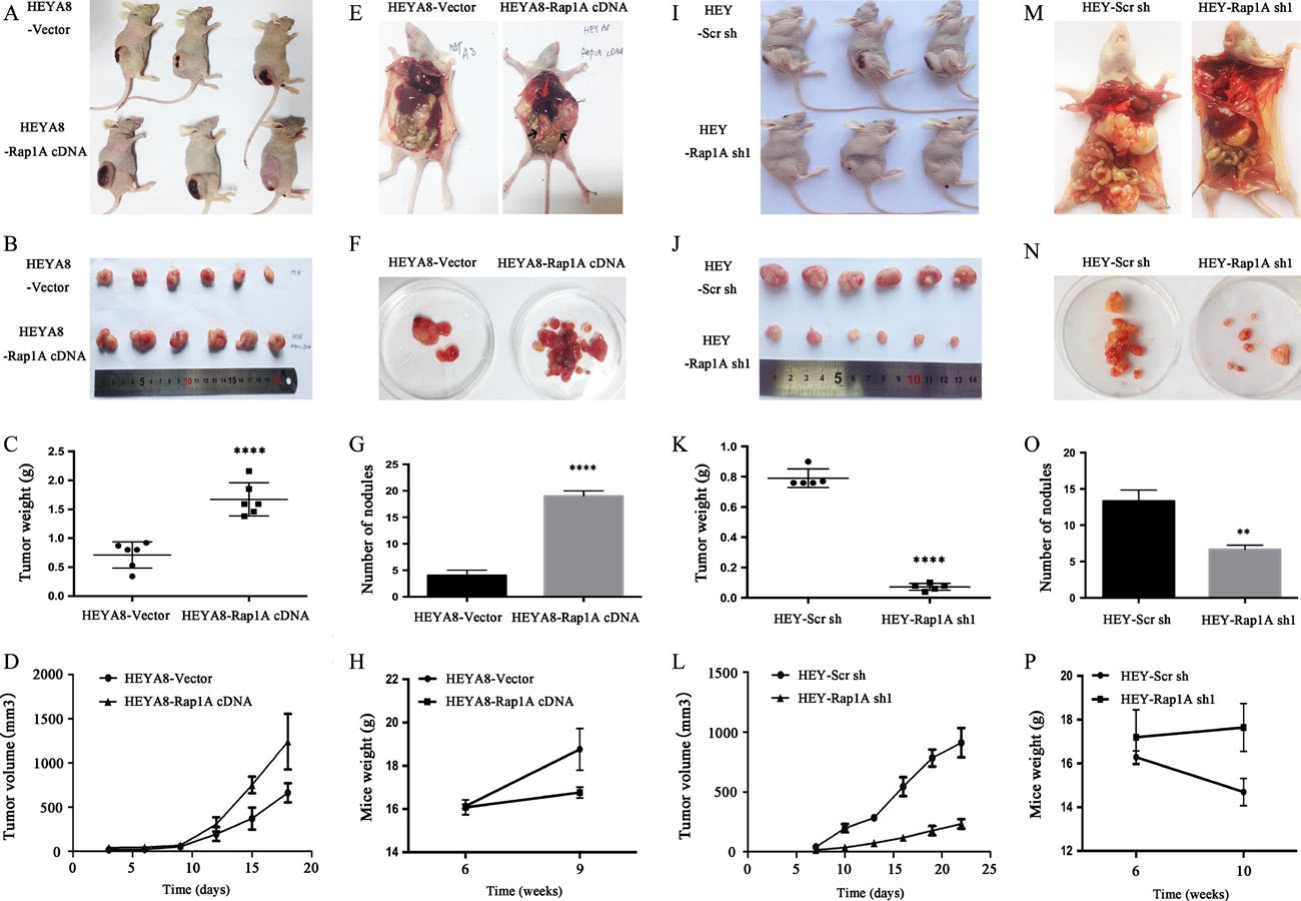
Rap1A accelerates tumorigenesis and metastasis. (A) Cancer cells were implanted subcutaneously at indicated numbers in 6‐week‐old nude mice. Mice were euthanized and necropsied after 17 days. (B, J) An illustrative diagram of extracted tumors belonging to the cancer cells injected mice and HEY‐Scr sh and HEY‐Rap1A sh1 celles**. (**C, K) An image of the weight of extracted nodules from one mouse's lung and colon. *n* = 6 mice/group**. (**D) Quantitative analysis of tumor volume with the time lapse from day 3 onwards. Error bars = 95% CIs. *n* = 6mice/group**.** (E) Intraperitoneally injection of cells into the 6‐week mice. Tumors were allowed to grow for 3 weeks and euthanized and necropsied. Black arrows show the lung and colon metastatic foci. (F,G and N,O) Upper panel: An image of extracted nodules from one mouse's lung and colon. Lower panel: an image of number of nodules. **P < 0.01. ****P < 0.0001 vs control; *n* = 6 mice/group. Mice weights were measured before being injected and euthanized, respectively. *n* = 6 mice/group**.** (H,P) Mice weights were measured before injected and euthanized, respectively. *n* = 6 mice/group. (I) Tumorigenicity assay: HEY‐Scr sh and HEY‐Rap1A sh1 cells were implanted subcutaneously at indicated numbers in 6‐week‐old nude mice. Mice were euthanized and necropsied after 22 days. (J) Quantitative analysis of tumor volume with the time lapse from day 7 onwards. Error bars = 95% CIs. *n* = 6 mice/group**.** (M) Metastatic assay: intraperitoneal injection of cells into the 6‐week mice. Tumors were allowed to grow for 4 weeks and euthanized and necropsied. Black arrows show the lung and colon metastatic foci.

To determine whether Rap1A contributes to ovarian cancer tumorigenesis and metastasis in vivo, we performed animal assays with cancer cells expressing Rap1A shRNA or Rap1A cDNA. Mice were subcutaneously or intraperitoneally injected with the above cells respectively. Tumor sizes were measured every 3 days. As shown in Figure 4, HEYA8‐Rap1A cDNA cells exhibited an increased tumor size compared with control cells (Fig. 4A, B, C, D). In addition, the number of metastatic nodules in lung or colon tissues from the xenografts animals injected with HEYA8/Rap1A cDNA cells was about fivefold (Fig. 4E, F, G) more than that formed in same tissues of animals injected with control cells. Importantly, we weighted the nude mice before being sacrificed and found that the mice treated with Rap1A cDNA expressing cells lost more weight compared with mice treated with control cells (Fig. 4H). Conversely, HEY‐Rap1A sh1 cells exerted a weaker effect on the tumor‐initiating ability than control cells (Fig. 4I, J, K, L). Accordingly, the mice intraperitoneally injected with Rap1A shRNA expressing cells had a reduced metastasis tumor by at least one order of magnitude (Fig. 4M, N, O) and with a higher weight than control mice (Fig. 4P). Together, these observations demonstrated that Rap1A exerts a strong effect on the tumor‐initiation and metastatic ability of ovarian cancer cells.

### Rap1A activates the ERK/P38 signaling

**Figure 5:**
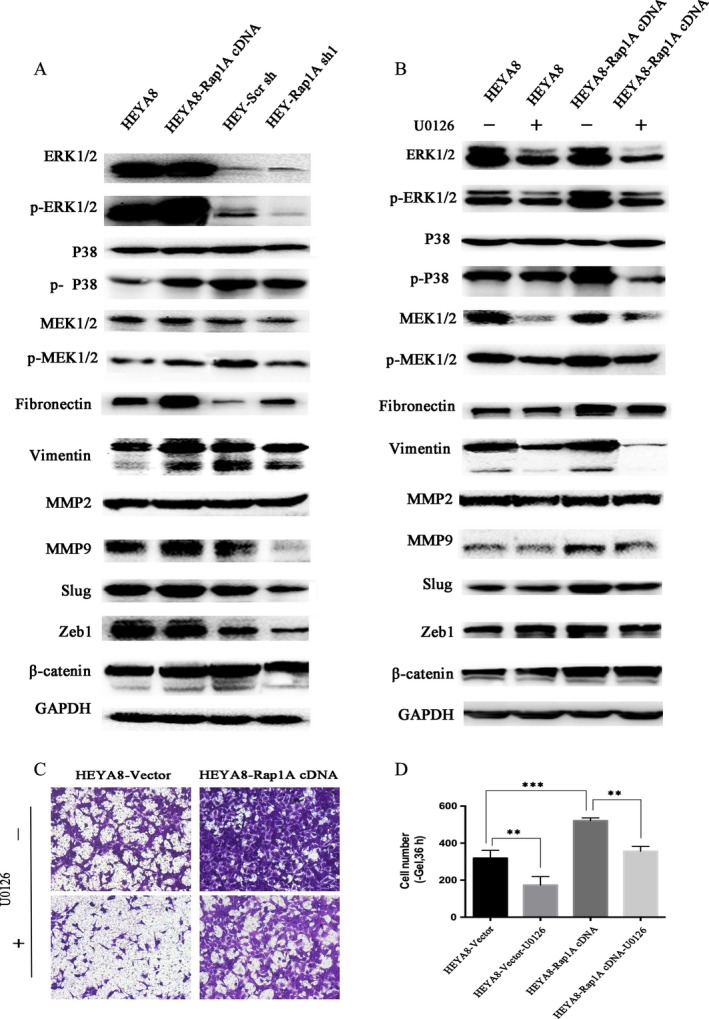
Rap1A regulates the mitogen‐activated protein kinase (MAPK)/ERK/P38 pathway. (A) Analysis of indicated proteins in cancer cells. GAPDH is a loading control. (B) Analysis of indicated proteins in HEYA8‐vector and HEYA8‐Rap1AcDNA cells in the presence or absence of DAPT at the 10 umol concentrations for 24 h. GAPDH is a loading control. (C) Microscopy images of migrated cells by cancer cells and their controls after incubation in permeable supports for 36 h, staining with crystal violet (magnification, 10×). (D) Quantification of migrated cells. (**P < 0.01 vs. the control; ***P < 0.005 vs. the control.). Error bars = 95% CIs. *N* = 3 wells (magnification, 10×).

To further identify the mechanism, we detected the changes of mitogen‐activated protein kinase (MAPK)/ERK and EMT associated signal molecules. As shown in Figure 5, upregulation of Rap1A induced an augment in the protein expression of phosphorylated p38, MEK1/2 and ERK1/2. Whereas, knockdown of Rap1A in Hey cells yielded the opposite results compared with control cells. Similarly, the protein levels of the EMT markers such as fibronectin, vimentin, MMP9, slug and zeb1 were increased remarkably in HeyA8‐Rap1A cDNA cells while interruption of Rap1A downregulated fibronectin, vimentin, MMP9, slug and zeb1, indicating that Rap1A may promote cancer cell migration and invasion through EMT (Fig. 5A). To strengthen the above results, we treated cells with the MEK inhibitor U0126 and detected the above proteins. U0126 treatment reversed the Rap1A overexpression‐induced increase of MEK1/2 and ERK1/2 both in phosphorylated and non‐phosphorylated levels. In addition, p‐p38, fibronectin, vimentin, MMP9, slug and zeb1 were also inhibited by U0126 (Fig. 5B). The transwell assay was also performed to show that the ovarian cancer cell migration ability was absolutely blocked after the addition of U0126 at 10 *μ*mol/L for 24 h(Fig. 5C,D). These data suggest that the MAPK/ERK/P38 signaling cascade may participate in the Rap1A‐driven cancer cell aggression and migration.

**Figure 6:**
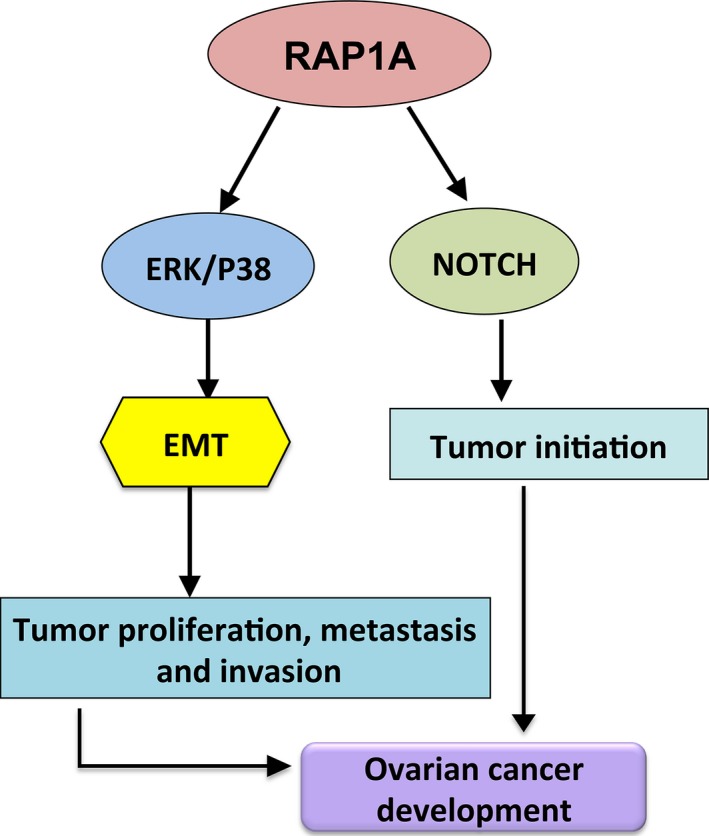
A schematic diagram showing the role of RAP1A in regulation of tumor metastasis capacities. The expression of RAP1A in ovarian cancer activates extracellular signal regulated kinase (ERK)/P38 pathway to promote EMT process and then enhances tumor proliferation, metastasis and invasion. NOTCH pathway is recruited to induce tumor initiation. EMT, epithelial‐mesenchymal transition.

## Discussion

In this study, we first investigated the function of Rap1A in ovarian cancer cell proliferation, migration and invasion through up‐regulation or down‐regulation of Rap1A. Our findings revealed that Rap1A overexpression enhances ovarian cancer cell metastasis and tumorigenesis, which is similar to those reported in several other cancer types 11, 12, 23.

However, the in vitro data we found are controversial to those reported by Lin et al. 24, in which they showed that Rap1A had no effects on ovarian cancer cell migration and invasion. This may be due to that they used fewer cell lines and generated insufficient data. Using 3D culture, we further evaluated the cell metastasis and invasion through observing different sizes and numbers of spheroids, which was reported by other scientists 25. Significant increase of spheroids was found in Rap1A overexpressing HeyA8 cells (Fig. 3A, B), while silencing of Rap1A or treatment with the Notch inhibitor DAPT (*γ*‐secretase inhibitor) reduced cell migration and tumor sphere formation (Fig. 3C, D, E, F, G). Literatures have reported that the tumor initiation is activated through the Notch pathway 26, and that activation of Notch signaling plays a significant role in ovarian cancer cell proliferation 27, while HES1, a member of the basic helix‐loop‐helix family of transcription factors, may be activated at the downstream of Notch 28. Our data showed the diminished expression of cleaved Notch1, 2, 3, Hes1, and Fibronectin in both HEYA8‐vector and HEYA8‐Rap1A cDNA cells following treatment with DAPT compared with the DMSO‐treated controls (Fig. 5B). Since the Notch induced EMT could contribute to tumor initiation according to literature 29, we supposed that the overexpression of Rap1A might stimulate the Notch activated EMT, which requires further research for specific mechanism. Besides the in vitro findings, our data also illustrated that Rap1A facilitates the tumorigenic and metastatic behaviors of ovarian cancer cells in vivo.

Since we found that Rap1A affected ovarian cancer cell proliferation, migration and invasion that were usually regulated by MAPK and EMT associated molecules 30, 31. We analyzed the main molecules involved in the MAPK/ERK/p38 pathway and EMT process, and found that Rap1A may induce EMT through the activation of MAPK/ERK/P38 signaling. Additionally, MMP2 and MMP9 are reported to be associated with cancer cell metastasis 32, while in ovarian cancer tissues, the increased MMP9 was associated with a higher risk of death, but no link was found between death or progression and MMP2 expression 33. We found that the expression of MMP9 but not MMP2 was markedly altered in both Rap1A overexpression and silencing ovarian cancer cells. Thus, Rap1A may promote ovarian cancer metastasis at least partially through the up‐regulation of MMP9.

In summary, our study provided clear data to show that Rap1A promotes ovarian cancer cell proliferation, migration and invasion via the ERK/p38 and Notch signal pathways (Fig. 6).

## Conflict of Interest

None.
